# Lectures on Hysteria and Allied Vaso-Motor Conditions

**Published:** 1910-03

**Authors:** 


**Lectures on Hysteria and allied Vaso-Motor Conditions.** By T. D. Savill, M.D. Pp. xiii, 262. London: Hy. J. Glaisher. 1909.

Whilst reviewing this book the premature death of the author was announced, and we therefore take this opportunity of offering our sincere condolences to the partner of his labours and collaborator in this work, Dr. Agnes Savill, in her sad bereavement. In this case it is not necessary to recall the aphorism "de mortuis" in expressing appreciation of the author's observations, for their intrinsic worth is their very sufficient commendation. The book consists of lectures published at various intervals from 1896 onwards, and herein gathered into a complete presentation of the author's views on hysteria. As is inevitable in lecture-form, there are repetitions, but the gain in vividness of presentment atones for occasional redundance. Throughout the work presents evidence of close thought upon the difficult problems of hysteria and of a sufficient clinical experience. As a matter of fact, its clinical basis rests upon 500 carefully-studied cases. Whilst the theories of other observers are fairly and lucidly represented, the main thesis of the book is the author's belief that vaso-motor disturbances play the chief part in the direct causation of hysterical disturbances, and that the chief defect is in the sympathetic nervous system. He certainly brings forward much evidence in support of his view, and the sections which deal with the vaso-motor cutaneous manifestations in hysteria are much to the point, and show his gift for original clinical observation. For these alone the book deserves study. Perhaps he pushes this theory of the origin of hysteria sometimes further than we can go with him, but he is at the same time careful to point out that no one explanation is sufficient for the protean phenomena of the disease.

Like other authors, he has to concede the existence of an hysterical predisposition on which as a basis the determining causes work. Is not this concession fatal to any knowledge of the true nature of hysteria? It is just this hysterical disposition that we either require to know the exact nature of, or, on the other hand, to be able to finally consign to the limbo of dead theories. After all, the impression that remains after the perusal of an able work like this is, that the true nature of hysteria has even now eluded all the theories that profess to account for it.

We conclude by recommending the work as a very interesting and lucid account of the disease, especially excellent upon the clinical side, marked here by much originality of observation, sound in the advice given as to treatment, and a worthy memorial of an accomplished physician.

